# The Surface Anatomy of the Abdominal Viscera

**Published:** 1901-08-24

**Authors:** Seymour Taylor

**Affiliations:** Physician at the West London Hospital


### Hospital Clinics and Medical Progress.

### THE SURFACE ANATOMY OF THE ABDOMINAL VISCERA.

Being the abstract of a Lecture delivered in June, 1901, by Seymour Taylor, M.D., F.R.C.P., Physician at the West London Hospital.

*(Concluded from page 328.)*

In mapping out the large organs of the abdominal cavity, it is perhaps best to begin with the liver. The greater mass of this gland is situated under the lower ribs, and if we take three lines of longitude, namely, one passing vertically through the nipple, another the mid-axillary, and the third the line of the lower angle of the scapula, and shall be guided in our marking out of the upper border of the liver. It rises as high as the fifth intercostal space in the nipple line, the seventh-costal space in the mid-axillary line, and the ninth space in the line ot the angle of the scapula. The lower border of the liver corresponds practically to the costal arch and should not be felt below it, but in the epigastric region itself the liver runs transversely across the middle line and its lower border extends to a point midway between the xiphoid cartilage and the umbilicus. The gall bladder may sometimes be felt if unduly distended at about the edge of the ninth costal cartilage.

The spleen in health should not be felt in the abdomen, except perhaps in thin emaciated children. Its long axis runs downwards and a little inwards, lying against the inner surface of the ninth, tenth, and eleventh ribs. If the organ is enlarged, it is projected downwards and a little inwards, and then the anterior border can be felt, as a sharp ridge with its typical notch.

The stomach in its normal condition is situated entirely on the left side of the body. Its curves have been called upper and lower respectively. But I would submit to you that this conveys an erroneous notion as to the position of the organ. The so-called upper curvature is much more vertical than you would imagine, and beginning on the left side of the lumbar vertebræ it runs downwards and a little to the right, and terminates at the pylorus, which is situated on the body of the second lumbar vertebra. The greater or external curvature varies considerably according to the distension of the organ, but it certainly runs more transversely across the abdomen than does the lesser curve, and it can be easily seen, on opening the abdomen, to occupy about three fingers' breadth of the space which is between the lower edge of the liver and umbilicus.

The kidneys lie deep in the abdomen on the quadratus lumborum muscles. They occupy roughly the space beneath the last rib and the crest of the ilium. They are embedded in a capsule of suet or fat, which helps to retain them in their place. In emaciated women, the fat having become absorbed, only an envelope of areolar tissue will remain, and this is insufficient to keep the organ in its place, and hence we have the movable kidney, notwithstanding the fact that the peritoneum lying in front of the organ must be supposed to keep it in its place. The movable kidney, although an abnormal condition in man, would appear to be a usual condition in the sheep, as anyone can observe for himself by inspecting the carcase of a sheep as it hangs suspended by the hocks from a butcher's shop. Here you will note that the organ hangs downwards as a pendulous tumour lying upon the diaphragm. In the emaciated sheep the kidney can be actually felt by thrusting the hand deep into the anterior abdominal walls. In the human subject the colon lies upon the kidneys; the ascending colon being in front of the right kidney and the descending colon in front of the left. These are perhaps the most important relations, although the right kidney has also part of the duodenum lying on it. It is obvious from what I have told you that surgical procedures upon this gland are best performed through the loin, the kidney being thus exposed from behind and separated from its capsule without injuring the peritoneum.

The small intestines are suspended to the backbone by the mesentery, which is a long fold of peritoneal covering as compared with the similar attachment of the great intestine. The consequence of this is that the small intestine is very mobile. This is important, as incised wounds of the abdominal parietes may cause the escape of part of the small intestines through the wound unless the wound is directly over the situation of the colon; the great probability is that the protruded gut will be small intestine with which you have to deal. The large intestine commences with the cæcum, which is not always constant in position. It occupies the right iliac fossa above the outer half of Poupart's ligament, but it may be variable in position, as I have said. It is almost completely enveloped with the peritoneal covering which forms a distinct mesocæcum.

The appendix itself has a still more variable situation. It may lie parallel with Poupart's ligament, or it may be directly underneath the cæcum, but for the most part the free end will lie on a line drawn from the anterior superior spine to the umbilicus. The valve which guards the entrance of the ileum into the cæcum is probably the most perfect valve in the whole body. I doubt if regurgitation of the contents of the large intestine is possible through this valve into the ileum. From the few experiments which I have made of injecting water under strong pressure from the rectum upwards, I have never been able to break down the resistance of this valve. I have found the intestine itself would burst before this valve would give way. The large intestine itself is approximately six feet long, and is divided, as you know, into ascending, transverse, and descending colon, followed by the sigmoid flexure and the rectum. The colon is considerably shortened in length by being pinched in as it were by the three longitudinal sets of muscular fibres. These fibres alone will enable you to detect the colon in any operation of the abdominal cavity, but in addition you have lumps of adipose tissue known as the appendices epiploïcæ in the peritoneal coat. These two peculiarities are absolutely distinguishing features of the colon. The ascending colon passes upwards lying on the right kidney to the under surface of the liver, where it makes a sudden bend downwards, again forming a distinct loop, and then coming to the front of the abdomen passes across the cavity in a line about two fingers' breadth above the umbilicus. It then passes up into the left hypochondrium and comes in intimate relation with the spleen, subsequently running down over the left kidney to the left iliac fossa, where the intestine now takes the name of sigmoid flexure. The sigmoid flexure itself varies considerably in different individuals in length and in position. In some it only goes to the middle line of the body; in other subjects it passes entirely to the right side before it turns back to the mid-line of the sacrum and becomes rectum. I can give no reason for the curved part of this bowel or for the variation and extent of its curvature, unless it be that by holding up the more solid fæces, it so prevents the undue desire for their propulsion until an opportune moment; but this I repeat is mere speculation. The rectum itself is a purely pelvic organ. It is only partially invested by peritoneum, but a knowledge of this investiture is important. The first portion is entirely covered by peritoneum; the second portion is only partially covered by peritoneum, whilst the third portion is entirely denuded of this coat. Now if you recollect that the third portion of the rectum is an inch long, that the second portion of it is only about three or four inches, and as I have said only the upper part covered by peritoneum, it follows that operations on the lower part of the gut must be conducted with care if you wish to avoid entering the serous cavity. Roughly speaking, in any surgical interference with the rectum three inches from the anus is about the limit of the field in which there is no danger of entering the peritoneal cavity.

In conclusion, it will be obvious to you all that I can only give you a cursory description of the principal relations of the important organs and structures in the thorax and abdomen, but if in the short time that has been at our disposal I shall have refreshed your memory of the anatomy of these organs, and if I shall have also given you any aid to diagnosis and treatment, my object will have been accomplished.

